# Economic feasibility analysis for an electric public transportation system: Two cases of study in medium sized cities in Mexico

**DOI:** 10.1371/journal.pone.0272363

**Published:** 2022-08-04

**Authors:** José Tomás Sánchez, Jesús Antonio del Río, Aarón Sánchez

**Affiliations:** 1 Instituto de Energías Renovables, Universidad Nacional Autónoma de México, Temixco, Morelos, México; UCSI University, MALAYSIA

## Abstract

This work presents an economic analysis that illustrates the feasibility and the possible benefits related to the replacement of internal combustion vehicles (ICVs)by electric vehicles (EVs) public transportation in medium-sized cities. According to the current operating conditions, we calculate the cost of operating internal combustion vehicles and compare them with a selected EV with approximately the same passenger capacity. We calculate the CO_2_ emissions in both cases. Moreover, for the case of EV, we analyze two scenarios: 1) Use the grid to charge the EV and 2) a grid-connected photovoltaic system using the available land in the store terminals. The net present value (NPV) indicates the feasibility of two EV replacement scenarios: EV fleet using energy from the grid and EV fleet with a PV system energy generation interconnected to the grid. The economic analysis considers the different prices of electricity according to the existing tariff schemes in Mexico. Due to the electricity generation mix in Mexico, in the case of CO_2_ emissions, the reduction is not as expected in the only grid connection; but a PV system reduces more than 30% CO_2_. This analysis was carried out for two medium-sized cities: Morelia, Michoacán, and Cuernavaca, Morelos, both in Mexico.

## Introduction

Climate change is the main challenge for humanity. Many countries, institutions and organizations are promoting and taking action to face this problem. The Paris Agreement has set the goal of holding the increase in the global average temperature to well below 2°C above pre-industrial levels and pursuing efforts to limit the temperature increase to 1.5°C above pre-industrial levels. The goals address forward carbon neutrality strategies as their long-term emission mitigation goals for the mid-21st century. So far, over 130 countries and regions have pledged themselves to net-zero or carbon neutrality targets, and 119 countries have submitted new or updated nationally determined contributions (NDCs) [1]. The transport sector contributes to air pollution and increases greenhouse gas emissions (GHG); it is at the same time a vulnerable sector to scarcity and the rise in the cost of fuels. The GHG emissions in Mexico from the transport sector, in 2015, were 171,355.53 Gg CO_2_, the auto transport sector contributed 93.3% of total emissions nationwide, that is 159,944 Gg CO_2_ [2]. Alternatives to decrease the negative impacts of the transportation sector, such as moving from internal combustion vehicles to no motor vehicles, public electric transport systems, like bikes, trains or trolleybus, are good options. However, the change to mobility based on these last options in small cities requires redesigning the cities’ infrastructure. Implementing more sustainable forms of transportation in urban spaces, such as walking and cycling, reduces energy consumption, pollution, and emissions, and provides health benefits. Active transportation plays a vital role in driving the change towards more sustainable mobility. Its health benefits reduce the risk of cardiovascular disease, type-2 diabetes or depression and can also reduce obesity levels [3]. Nowadays, in Mexico, as in many Latin American countries, public transportation systems use fossil fuels. In addition, the public transport service is provided mainly through old, inefficient, and polluting internal combustion vehicles. Since the possibility of electric mobility for passenger transport in Mexico has not been sufficiently studied, a significant change in public transport is not perceived in the short and medium-term. For this reason, it is essential to carry out this type of study in the context of developing countries, like Mexico, which explores the opportunity to move towards electric mobility. Electric transportation offers ideal opportunities for the broader introduction of renewables energies (RE) to the transport sector. As energy-consuming technologies, electric vehicles (EVs) create new demand for electricity, which (ER) can supply. In addition to the benefits of this shift, such as reducing CO_2_ emissions and air pollution, electric mobility also creates significant efficiency gains and could emerge as an important source of storage for variable sources of renewable electricity [4]. To maximize the benefits of electric mobility, a decarbonized energy supply, based on renewable sources, should be considered, otherwise CO_2_ emissions may even increase [5]. Because of the fallen down of prices for solar photovoltaic (PV) in the last decade, solar electricity in providing the energy for electromobility is appealing [6]. Many papers have addressed the issue of electric mobility. For instance Ruggieri et al. [7] analyzed air pollution decrease in six european cities (London, Hamburg, Oslo, Milan, Florence, and Bologna) with rapid EV development due to electric mobility policies, M.J Booysen et al. [8] studied electric vehicles with solar energy for sustainable informal public transport in Uganda, the results show that the median energy demand of the fleet of taxis was 220kWh with a median charging potential (stationary time) across taxis of 8h/d to 12h/d. The median potential for charging from solar pv ranged from 0.24 kWh/m^2^ to 0.52 kWh/m^2^ per day, across the taxis. Mariusz Kubanzki [9] analyzed the introduction of electric buses to common use in public transport in Czechowice- Dziedzice. He carried out a comparative analysis of the application of Diesel, Hybrid and electric buses in public transport in Czechowice- Dziedzice. The advantages and disadvantages were pointed out based on simplified cost calculation taking into account the cost of purchase and operation of buses. Yongxing Wang et al. [10] proposed a multistage optimization model to provide Battery Electric Vehicles (BEV) drivers with a charging strategy for intercity travel. The results from the study indicate that the charging time accounts for 10.32%-13.48% of the travel time. It is difficult to reconstruct the highway network to decrease the driving time for intercity travels. Therefore, this is necessary to reduce the charging time by adopting high power chargers along highways to improve the travel efficiency and then attract more drivers, especially the drivers with relatively high value of time, to complete intercity travels using BEVs. Nathan Parker et al. [11] estimate 5-year ownership costs for four EVs and four gasoline-fueled vehicles by neighborhood across Los Angeles County. Due to variation in electricity rates, insurance rates, and vehicle miles traveled, the median cost varies across neighborhoods by a factor of between 1.2 and 1.35, depending on the vehicle. Median ownership costs are usually higher for EVs than for comparable gasoline-fueled cars, but considering the entire distribution of vehicle miles traveled suggests that buying an EV saves over 17% of households. Marc Schmidt et al. [12] address the issue of fleet electrification by analyzing a data set of 81 empirical mobility patterns of commercial fleets. They conduct a simulation to design a decision support system for fleet managers evaluating which fleets have an adequate potential for electrification and how fleets can improve the number of successful electric trips by adapting their charging strategy. Mads Greaker [13] proposes a theoretical model covering both the entry of charging stations and the sales of EVs. The model is used to study policies for developing a good charging network for existing and future EV owners. Chandra Mouli et al. [14] analyzes the direct charge of electric vehicles using a 10kWp grid-connected PV system, compares operation costs between ICV and EV, driving an EV instead of ICV generates an annual saving of 1,520€ and reduces CO_2_ emissions of 770kg/car/year for a distance of 20,000km. Moreover, zero-emission driving is possible using a 13kwp PV system; it can annually cost 70,300 km for Nissan Leaf and 49,620km for a Tesla Model-S. The cost of PV energy is 10c/kWh, which is less than half of the current grid price of 23c/kWh. Tang et al. [15] evaluated four scenarios for an EV fleet in Shenzhen, China: grid-EV, grid-PV-EV, PV-battery-EV, and grid-PV-battery-EV. The results show that PV-powered EVs can reduce GHG emissions significantly compared with grid-connected EVs. The carbon tax rate has a significant impact on the grid-EV scenario. These rates can be used as a policy tool to accelerate transportation systems that can generate low carbon emissions. On the other hand, an analysis of the feasibility of a solar parking lot for EVs in Lisboa shows that for the current market conditions, the payback time is 14 years; a public financial incentive would improve the project economics, making the payback drop to 7 years [16]; while Ellen de Schepper et al. [17] show the economic benefits of the direct coupling of solar electricity with EVs. Results show that it is most profitable to invest in EVs at low electricity prices (<€0.112/kWh). When the price of electricity rises (>€0.134/kWh), investment in exclusively, PV becomes most attractive. In all other cases, it is more profitable to invest in the combination of both technologies. Bedir et al. [18] use empirical data on residential energy consumption and photovoltaic generation to assess the effect of the combination of residence consumption, electric vehicle charging, and solar panels. Using an energy management system, the authors found that savings of 30 to 50% in electricity billing can be achieved. Wi et al. optimized the electric charge of 12 vehicles minimizing total electricity costs. Electric mobility was modeled with the time of arrival and departure. The smart charge defined by the generation of photovoltaic energy could reduce the purchase of energy from the grid by 6-15% compared to an uncontrolled load [19]. Van der Meer et al. [20] manage the electrical charge of 6 vehicles in a workplace equipped with a nine kWp photovoltaic system. The optimized charge improves self-consumption by 20%. Guo et al. [21] address a two-stage framework for the economic operation of a microgrid-like electric vehicle (EV) parking deck with on-site renewable energy generation (roof-top photovoltaic panel). First, an algorithm calculates the necessary daily grid energy and the station’s marginal cost. A second algorithm optimizes the charging time of electric vehicles. This strategy increases the photovoltaic charging station’s income by 3% in summer and 10% in winter. Seeding et al. [22] analyzed a 100kWp charging system to charge 650 electric vehicles. The authors explore the possibility of integrating solar PV energy through smart charging strategies of three different electric vehicle fleets—namely, commercial customers, commuters, and opportunity parkers.

A day-time photovoltaic based charging station is considered by Tulpule et al. [23]; the results show the impact of PV based workplace charging on economics and emissions from the power grid. The optimal control algorithms were applied to the parking garage showing that the optimization algorithms have less impact on the payback time of the parking garage but reduce CO_2_ emissions by 90% as compared to charging without solar electric power. Antti Lajunen [24] presents a cost-benefit analysis of hybrid and electric city buses in fleet operation; the results indicate that plug-in hybrid and electric buses have the best potential to reduce energy consumption and emissions. Matthias Rogge et al. [25] analyze how and to what extent existing bus networks can be electrified with fast charging battery buses. They found that 50% of the service trips can be electrified with a charging power capability of 300 kW and a usable battery capacity of 220 kWh. This electrification is possible even under worst-case conditions using currently available battery systems without changes in the existing schedules. P. Plötz [26] presents an overview of empirical findings on actual plug-in hybrid and battery electric vehicles (PHEV and BEV) for the USA and Germany. The results show that PHEVs with about 60 km of the real-world range currently electrify as many annual vehicles kilometers as BEV with a much smaller battery. In addition, the use of renewable energy can highly contribute to reducing greenhouse gas in car transport. Mario Mureddu et al. [27] study how renewable energy impacts infrastructures considering the full deployment of electric mobility. The analysis reveals long-range effects on infrastructures outside metropolitan areas and points out the most relevant unbalances by spatial segregation between production and consumption areas. Their results suggest adopting planning actions supporting the installation of renewable energy plants in areas mainly involved by commuting mobility, avoiding spatial segregation between consumption and generation areas. Yuhua Zheng et al. [28] analyze the effects of the introduction of EVs in China; Zheng et al. assess the energy-saving and emission-reducing impacts of the projected penetration of EVs until the year 2030. They define five scenarios of various EVs penetration rates; their results indicate reductions in transport GHG emissions and gasoline and diesel consumption by 3.0%–16.2%, 4.4%–16.1%, and 15.8%–34.3%, respectively, will be achieved by 2030 under China’s projected EV penetration scenarios.

In Mexico, the public transport service is granted to individuals by the government and none of them have ventured into or experienced the use of electric vehicles to provide public transportation services. We consider that the electrification of public transport is one of the needed actions towards sustainable cities. Two cities (Morelia and Cuernavaca) have been selected to determine the electric passenger transportation economic feasibility. This work shows the opportunity to consider the use of electric vehicles powered by photovoltaic solar energy for the public transport sector.

## Two specific cases of public transport in medium-sized Mexican cities

In Mexico, there are more than 50 cities with a population of between 300 thousand and a million people. Most of them are in the central region of the country around a latitude of 20°. We select two medium-sized cities with these features Morelia City and Cuernavaca City. Cuernavaca is the capital of the state of Morelos, it is located 85 km south of Mexico City and it has a population of 378,476 inhabitants [29]. Morelia is the capital of Michoacan, in central Mexico, is the largest and most populated city in the state of Michoacan with 849,053 inhabitants [30], and it is the most important city of the state from the social, political, economic, cultural, and educational points of view.

### Cuernavaca RUTA UNO

In Cuernavaca city, several companies provide urban and suburban public transport services covering various routes; within these, RUTA UNO is the one with the greatest distance traveled, it uses Mercedes Benz model Boxer 50 buses and has been taken as a case of study since it has an impact on fuel consumption and polluting emissions.

The public transport service begins at 6:00 hrs, and it ends at 23:00, vehicles leave in 5 minutes intervals. There are two stations denominated station A, and station B. Station A is located south of Cuernavaca city, in the “Guacamayas” area (18.8747, -99.2197). From this point, the buses travel 20 kilometers to get to station B, located north of the city (18.9773, -99.2357) in the area known as “Universidad”. The coordinates and the calculations of distance and surfaces were made using Open Street Maps. The economic feasibility of replacing ICVs with electric vehicles on Line ONE is presented later.

### Morelia GRAY ROUTE

In Morelia, the population travels mainly by motorized vehicles, within which public transportation covers almost 55% [31] of them. This service is provided by 15 different lines / routes that differ from each other by numbers and colors. Two types of vehicles are used to provide the service to the population, Nissan Urvan and Toyota Hiace. In this work we take as a case study the route known as GRAY ROUTE (RUTA GRIS). Gray route has two branches identified as ***C*** and ***R***, where R denotes “Realito” and C denotes “Camelinas”. Gray route circulates through one of the city’s main roads, and it is one of the lines that makes more displacements throughout the day, which has an impact on fuel consumption and polluting emissions. The Gray route has a single bus storage station located to the west of the city (19.6855,-101.2356). Vehicles leave the station and circulate 30 kilometers through the periphery of Morelia city. The public transport service is available from 6:00 hrs to 23:00, vehicles leave in 3 minutes intervals.

In Table 1, we resume the distances and trip times for both cites’ transport routes.

**Table 1:** Identified service trips for a workday.

Trips features.				
Route	Trip distance (km)	Trip time (min)	Gap (min)	Distance (km)
Camelinas	30	60	3	510
Realito	30	60	3	510
RUTA UNO	20	70	5	160

We have presented the features of these two transport services for both cities. Our starting point is considering that by changing the ICV by EV, the transport services will diminish the *CO*_2_ emissions. It is essential to mention that we consider the electrical distribution grid as unlimited energy storage and supply source, and we assume that the grid capacity is enough to meet the EVs demand. In addition, we analyze CO_2_ emissions from ICVs and EVs fleets. In the next section, the methodology we use to perform our analysis is presented. In the section, economical feasibility of the use of EVs in medium-sized Mexican cities, we present our main findings and discuss them. Finally we close the paper with some remarks.

## Calculation procedure

**Fig 1:**
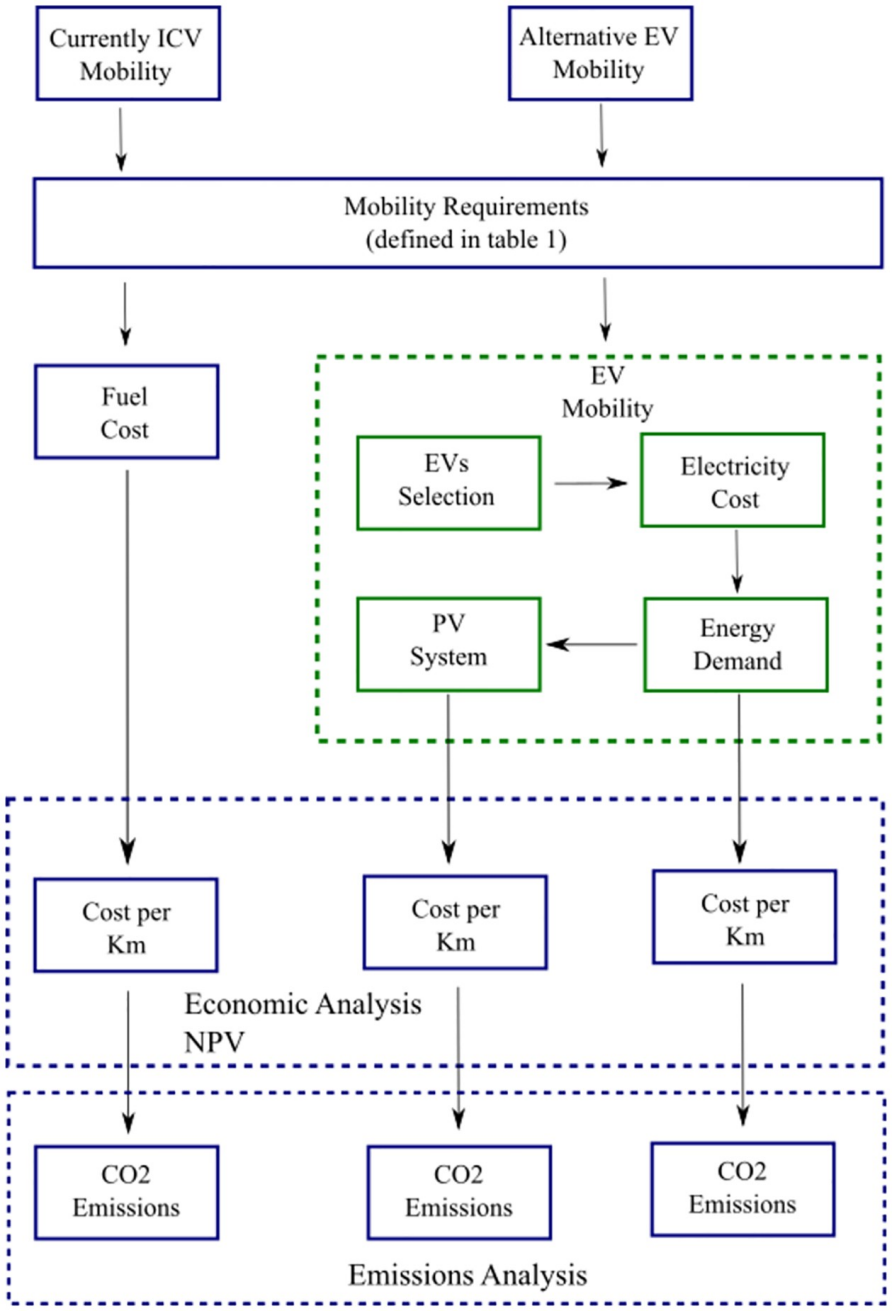
This block diagram illustrates the calculation procedure used in this work. The arrows indicate the following steps.

This section details the methodology we use to determine the fuel cost for the current ICVs and present it in an annual amount. We explain the manner that Mexico is calculated the electric tariff according to the electrical demand. In order to compare, we calculate the cost per kilometer in both cases, ICV and EV. Also, we describe our criterium of selecting the electric vehicles in correspondence with the used vehicles. Moreover, we present an analysis varying the manner to obtain the electricity to charge the EV’s by a) interconnection to the grid or b) using the available surface in the store terminals to install PV systems and contribute with solar energy to the electricity demand. Furthermore, we carry out an analysis of CO_2_ emissions in which we compare the emissions of the current transportation system with the emissions of the proposed electric mobility. Fig 1 is a diagram illustrating the methodology used.

### Fuel costs

Fuel cost are determined by the fuel price, travel distance, and vehicles performance. Fuel prices in Mexico are regulated by the Energy Regulatory Commission (Comisión Reguladora de Energía, CRE) [32]. There are two types of gasoline in Mexico: Magna and Premium. Magna gas is the most used by drivers, especially in public transportation, because it is cheaper than Premium. Magna gasoline is equivalent to Regular unleaded, and Premium gasoline is similar to Premium in the international market. Its main feature is its octane level: Premium has 92, and Magna has 87.

The cost per kilometer in an internal combustion vehicle (ICV) is given by:

$$\begin{array}{c} C_{Km}=\frac{F_{p}}{P}, \end{array}$$ (1)

where *C*_*km*_ is the cost per km, expressed in USD/km, *F*_*p*_ is the fuel price, *P* is the vehicle performance km/l.

Annual fuel costs are calculated according to the following expression.

$$\begin{array}{c} C_{A}=C_{Km}D \end{array}$$ (2)

where *C*_*A*_ is the annual fuel cost, expressed in USD, *D* is the annualy distance traveled by a vehicle expressed in km.

#### Total Annual Cost

According to information provided by the gray line and ruta uno managers, “minor” maintenance services are carried out every three months on vehicles, which consists of replacing spark plugs, oil, air filters, etc. We consider a typical average maintenance cost of $180 USD for Toyota Hiace vehicles, $155 USD for Nissan Urvan and $250 USD for Mercedes Benz Boxer. These maintenance costs are added to the fuel costs; therefore, the total annual costs are given by:

$$\begin{array}{c} TAC=C_{M}+C_{A_{n}} \end{array}$$ (3)

where *TAC* is the total annual cost, *C*_*M*_ is the maintenance cost per year, $C_{A_{n}}$ is the annual fuel cost in USD.

### Electric tariffs

In Mexico, Federal Electricity Commission (Comisión Federal de Electricidad, CFE) is the company that supplies the electric power service through the General Distribution Network(Red General de Distribución,RGD), belonging to the National Electric System (Sistema Eléctrico Nacional, SEN). The CRE is the office that sets the tariff schemes in the country. Energy costs are based on energy demand as well as the average ambient temperature of a region [33]. There are different rates and the average price per kWh according to different zones as described below.

#### Industrial tariff

Tariff OM or High Demand Medium Voltage (GDMTO) is applied to services that allocate energy to any use, supplied in medium voltage, with a demand of less than 100kW. HM tariff or High Demand in Medium Tension Time (GDMTH) is applied in medium voltage services for any use with a demand of 100kW or more. Due to energy demand, the tariff used in this analysis is the HM tariff.

#### HM tariff

This tariff has the characteristic of having different energy costs assigned according to the hour of the day and the season of the year. This hourly change in costs would allow us to determine the schedule strategy with the lowest energy cost to recharge the vehicles electrically. Tables 2 and 3 show energy prices depending on the time of day of the HM tariff.

**Table 2:** HM region central tariff.

HM tariff features			
*Day*	Base (0.05USD/kW)	Intermediate (0.06USD/kW)	Peak (0.12USD/kW)
*MondaytoFriday*	0:00-6:00	6:00-18:00 and 22:00-24:00	18:00-22:00
*Saturday*	0:00-8:00	8:00-19:00 and 21:00-24:00	19:00-21:00
*Sunday*	0:00-18:00	18:00-24:00	

Table 2 shows the schedules established by CFE for the HM rate from the last Sunday of October to the Saturday before the first Sunday of April.

**Table 3:** HM region central tariff.

HM tariff features			
*Day*	Base (0.05USD/kW)	Intermediate (0.06USD/kW)	Peak (0.12USD/kW)
*MondaytoFriday*	0:00-6:00	6:00-20:00 and 22:00-24:00	20:00-22:00
*Saturday*	0:00-7:00	7:00-24:00	
*Sunday*	0:00-19:00	19:00-24:00	

Table 3 shows the schedules established by CFE for the HM rate from the first Sunday of April to the Saturday before the first Sunday of October.

In addition to these costs, CFE charges fees for power capacity and distribution, $16 and $11 USD/kW in Cuernavaca city region and $17 and $8 USD/kW for Morelia city respectively [34]. These additional costs can be calculated from the following equation.

$$\begin{array}{c} Demand=\frac{Q_{m}}{24\times d\times FC} \end{array}$$ (4)

Here *Demand* is the amount of energy required by the user, *Q*_*m*_ refers to the monthly energy demand from the grid, 24 is the hours in a day, *d* means the number of days in a month, and *FC* is the charging factor (0.57 for HM tariff) which is provided by CFE and depends on the user tariff.

### Electric performance and cost per km

The battery performance parameter helps determine the lifespan of the storage system, but we are unable to obtain it from the manufacturer. Then, we assume that the specific energy of a Li-ion battery depends on the design and characteristics, but they range from 120–220 Wh/kg [35]. The lifespan of a battery in freight BEVs is around six years (1000 to 2000 deep cycle life) due to battery degradation [36]. Therefore, the battery’s capacity diminishes to 70–80% of the original value in this period [37].

The electrical performance can be established as the number of kilometers then an electric vehicle can cover, defined by range, with the amount of energy provided in its batteries.

$$\begin{array}{c} P_{elec}=\frac{Rng}{B_{C}}, \end{array}$$ (5)

where *P*_*elec*_ represents the EV electric performance, *B*_*C*_ corresponds to battery capacity expressed in kWh, and *Rng* is the EV range expressed in km.

According to Eq 5, the E5 Shiyan Li-Ion battery electric performance results in 3.4km/kWh, that means that 1 kWh is needed to travel a distance of 3.4 kilometers. Haojing’s Li-Ion electric performance is 4.1 km/kWh. The EV cost per kilometer depends directly on the price of the energy and the electric performance. It is given by:

$$\begin{array}{c} C_{{km}_{elec}}=\frac{P_{energy}}{P_{elec}} \end{array}$$ (6)

where $C_{{km}_{elec}}$ is the EV cost per km in USD/km, *P*_*elec*_ represents the electric performance in km/kWh, and *P*_*energy*_ corresponds to the energy price provided by the supplier (CFE) in USD/kWh.

Annual cost is obtained according to:

$$\begin{array}{c} CA_{elec}=C_{{km}_{elec}}D \end{array}$$ (7)

where $C_{{km}_{elec}}$ is the EV cost per km in USD/km, and *D* represent the annual distance traveled by the EV in km.

### Required energy

Although in many cities, the dissipative heat from the IC engine is used to warm the interior of the buses; since the average minimum temperatures in winter in the case study cities are around 15°C and the average temperature is around 25°C [38], the standard ICV used today has no heating system. We have not considered it in the calculations. Another point for the electrical consumption is the door opening/closing system. We have looked for a typical opening/closing system consuming 120W/12VDC and opening/closing time of 2.5 seconds [39], and then the energy consumption would be around 0.08Wh. We assume that the use of this auxiliary system does not contribute considerably to the discharge of the electric vehicle battery.

Energy demand is given by:

$$\begin{array}{c} E_{D}=\frac{1}{P_{elec}}\times D\times N_{EV}, \end{array}$$ (8)

here *E*_*D*_ is the energy required for the EV in kWh, *P*_*elec*_ is the EVs performance in km/kWh, *D* represents the distance traveled by the EV in km, and *N*_*EV*_ is the number of electric vehicles.

### Grid connected photovoltaic systems

We select a photovoltaic system connected to the grid to provide a fraction of the energy required by electric vehicles and reduce energy demand from the grid.

#### PV system energy production

The PV system energy production depends directly on solar resource, the ambient temperature, and the electrical efficiency of the system, and it is given by the following expression:

$$\begin{array}{c} E_{g}=P_{P}\times R_{S}\times R_{t}\times \eta_{t}. \end{array}$$ (9)

In this equation, *E*_*g*_ defines the PV system energy production, *P*_*P*_ is the PV system peak power, *R*_*S*_ represents the solar resource, *R*_*t*_ denotes the thermal performance and *η*_*t*_ is the electrical efficiency of the wiring and inverter.

#### PV sizing

For each study case, two criteria were applied to sizing photovoltaic systems; energy requirement and available area in land owned by the line’s administration. Energy balance suggests that:

$$\begin{array}{c} E_{g}=E_{TC}, \end{array}$$ (10)

here *E*_*g*_ is the energy generated by the photovoltaic systems and *E*_*TC*_ is the quantity of energy required by the electric vehicles.

Then, photovoltaic power is given by:

$$\begin{array}{c} P_{SPV}=\frac{E_{TC}}{R_{t}\times R_{s}\times \eta_{t}} \end{array}$$ (11)

Regarding available surface criteria, the maximum power of the photovoltaic system follows the next expression, which considers global irradiance *G*_*p*_(1000 W/m^2^), active surface *S*_*A*_ in m^2^ and the module efficiency *η*_*pvm*_.

$$\begin{array}{c} P_{p}=G_{p}\times S_{A}\times \eta_{pvm} \end{array}$$ (12)

### Photovoltaic energy required by electric mobility

The amount of renewable energy required for the operation of the selected electric vehicles would be as follows:

In order to calculate the energy that the photovoltaic system can produce, it is necessary to determine the power losses associated with the effects of the ambient temperature on the photovoltaic modules. Considering the temperature coefficient (*C*_1_) of the selected module, the irradiance (*G*), the ambient temperature (*T*_*amb*_) and the temperature of the module (*T*_*m*_), the power losses were determined. The module temperature is given by:

$$\begin{array}{c} T_{m}=T_{amb}+C_{1}G, \end{array}$$ (13)

$$\begin{array}{c} C_{1}=\frac{\left(T_{NOCT}-T_{amb}\right)}{G_{NOCT}}, \end{array}$$ (14)

where *T*_*NOCT*_ is the PV module temperature under Normal Operating Cell Temperature conditions (°C), *T*_*amb*_ is the ambient temperature for *NOCT* conditions (20°C) and *G*_*NOCT*_ is the irradiance for same conditions (800W/m^2^).

Once the module temperature is known, the temperature differential is estimated as:

$$\begin{array}{c} \Delta_{T}=T_{m}-T_{STC}, \end{array}$$ (15)

*T*_*STC*_ is the temperature at standard conditions (25°C)

Power losses are given by:

$$\begin{array}{c} P_{L}=C_{PMAX}\times \Delta_{T} \end{array}$$ (16)

*C*_*PMAX*_ is the maximum power temperature coefficient of the selected photovoltaic module expressed as a percentage per grade centigrade.

Thermal efficiency (*R*_*t*_) is calculated with power losses (*P*_*L*_) associated with the temperature:

$$\begin{array}{c} R_{t}=100-P_{L} \end{array}$$ (17)

The efficiencies of the inverters and wiring are considered constant [40]. The data of the ambient temperature and solar resource in the cities were obtained from the NASA meteorological base [41].

### CO_2_ emissions

We calculate the CO_2_ emissions produced by ICVs according to IPCC methodology [42]. Afterward, we compare CO_2_ emissions by EVs operation. Our emissions analysis considers the emission factor of the National Electric System (SEN) issued by the (CRE) [43], and the emission factors for fossil fuels provided by the National Institute of Ecology and Climate Change (Instituto Nacional de Ecología y Cambio Climático, INECC) [44]. We present the emissions factors in the Table 4.

**Table 4:** Electricity, Diesel, premium and magna gasoline emission factors.

CO_2_ Emission factors.			
Premium	Magna	Diesel	Electricity
2.304	2.344	2.599	0.582

For Premium, Magna and Diesel, emission factor units are (kgCO_2_/l), for electricity is (TON CO_2_/MWh).

To determine ICVs emissions, we consider the ICV performance (L/km). Nissan and Toyota performance are shown in the Table 5.

**Table 5:** ICVs Toyota, Nissan and Mercedes Benz performance.

ICV Performance.		
Nissan Urvan	Toyota Hiace	Mercedes Benz Boxer
0.140	0.149	0.353

ICV performance is expressed in liters of fuel per kilometer.

Applying the following expression we determinate ICVs CO_2_ emissions.

$$\begin{array}{c} E_{ICV}=D\times P\times EF \end{array}$$ (18)

where *E*_*ICV*_ refers to ICVs CO_2_ emissions, *P* denotes the ICV performance in liters per kilometer (L/km) and *D* is the distance traveled by the ICVs in km, finally *EF* corresponds to emission factor (premium, magna or diesel).

The EVs emissions due to the electricity generation for its operation are calculated with the following expression.

$$\begin{array}{c} E_{EV}=E_{D}\times EF_{SEN}. \end{array}$$ (19)

In this equation *E*_*EV*_ refers to the EVs operation CO_2_ emissions, *E*_*D*_ is the energy demand from the grid by the EVs expressed in MWh, once and *EF*_*SEN*_ is the emission factor of the national electric system expressed in TON CO_2_/MWh.

#### Emissions’ reduction

We analyze the reduction in CO_2_ emissions due to the use of solar energy and EVs. The proposed photovoltaic system produces a renewable energy fraction per year of the energy required by EVs.

$$\begin{array}{c} F_{R}=\frac{E_{PV}}{E_{D}}. \end{array}$$ (20)

Here *F*_*R*_ is the energy fraction of renewable energy expressed in percent, *E*_*D*_ represents the EVs energy annual demand in MWh, and *E*_*PV*_ is the annual energy produced by the PV system expressed in MWh.

Hence the *CO*_2_ emissions regarding solar energy are given by:

$$\begin{array}{c} E_{EV-GR-PV}=E_{EV}-\left[F_{R}\times E_{EV}\right] \end{array}$$ (21)

In this expression, *E*_*EV*−*GR*−*PV*_ are the CO_2_ emissions due to the use of solar PV energy, *E*_*EV*_ are the *CO*_2_ emissions generated using energy from the grid and *F*_*R*_ is the fraction of renewable energy generated by the PV system.

### Economic analysis

In this section, we perform a cost comparison between ICV vehicles versus electric mobility. Afterward, we establish and evaluate two economic scenarios for electric public transportation. The first one contemplates the use of energy from the grid (Grid-EV). The second scenario involves the energy contribution from the proposed PV grid-connected system (Grid-EV-PV). To compare the economic performance of Grid-EV and Grid-EV-PV options, we use Present Net Value (NPV) as a financial indicator to determine the viability of each scenario. NPV is calculated according to the following equation:

$$\begin{array}{c} NPV=\frac{TAC[{\left(1+i\right)}^{n}-1]}{i{\left(1+i\right)}^{n}} \end{array}$$ (22)

In this expression, *TAC* is the total annualized cost in USD, *i* is the annual interest rate, and *n* = 25 expresses the number of years.

In this study, we have considered EV cost, energy cost, fuelling cost, maintenance and repair costs, PV cost [45] and cost of battery replacement. We have used the cost of battery replacement provided by BloombergNEF [46], and the energy produced by the photovoltaic system.

In the next section, we describe our findings to the substitution of ICV’s by EV’s in the transport system of two medium-sized Mexican cites.

## Economical feasibility of the use of EVs in medium-sized Mexican cities

This section presents our findings on the economic feasibility of using EVs in public transport in the specific cases of Cuernavaca and Morelia Cities. First, we present the EV selection for each city. Second, we present the required energy to determine the PV system dedicated to charging the vehicles. Later, we calculate the avoided *CO*_2_ emissions by using electric vehicles instead of ICV. Finally, we present the economic analysis of comparing ICV with EV in two scenarios: electric mobility with energy supplied entirely by the grid (Grid-EV), and electric mobility with the contribution of a photovoltaic energy generation system (Grid-EV-PV).

### Electric vehicle selection

For the Morelia city case, we selected an Electric Van type vehicle (EV) model E5. This electric vehicle is manufactured in China by the Rockrich company. Its dimensions are similar to the Nissan, and Toyota internal combustion vans provide public transport service today [47]. Because in Cuernavaca city public transportation service is provided by buses, an electric bus (Ebus) made in China by Haojing International Trade with similar passenger capacity was chosen [48]. The two different models of electric vehicles are equipped with 88kWh (Model E5) and 60kWh (Model Haojing) Li-Ion batteries, respectively. By selecting these electric mobility alternatives, we would not significantly alter the mode of operation during transition. In this section, we perform an economical and comparative analysis taking into account its technical characteristics. In Table 6, we present the main features of the selected EV.

**Table 6:** Main characteristics of the selected electric vehicles.

EV selected features.						
*City*	Vehicle Model	Battery (*kWh*)	Range (*km*)	Price (*USD*)	Curb Weight (*kg*)	Motor Power (*kW*)
Morelia	EV Van E5	88	300	$45,000	2000	45
Cuernavaca	Ebus Haojing	60	250	$68,000	5100	50

Table 6 shows the main features of the EV selected for each city, this information is provided by the manufacturer.

The GRAY ROUTE operates with 42 units, traveling daily 21,420 km; each selected electric wagon requires 145kWh to cover that distance; and 2,293 MWh would be required annually. In Cuernavaca city, RUTA UNO operates with 76 Mercedes-Benz model Boxer buses, each bus travel 160 km daily, it has a total distance of 12,160km, thus 1,065 MWh would be required annually. We present the required electric energy in Table 7.

**Table 7:** Required energy by Electric Vehicles proposed.

EV Selected Energy Demand.				
*City*	*EVs*	*Distance*(*km*)	*EnergyDemand*(*kWh*/*km*)	*AnnualEnergy*(*MWh*)
Morelia	42	21,420	0.29	2,293
Cuernavaca	76	12,160	0.24	1,065

Table 7 shows the energy demand associated with the replacement of the entire current fleet of internal combustion vehicles by the selected electric vehicles according to electrical performance and required kilometers.

### Peak power

Considering the actual land features of the available spaces in the actual garage vehicle of both routes, we establish as the best alternative to install photovoltaic panels composed of two and three photovoltaic modules placed in a vertical and horizontal position, having a capacity of 1,104 in Morelia city and 809 photovoltaic modules in Cuernavaca City. LG model NeONRLG monocrystalline photovoltaic module [49] with a peak power of 370W was selected. So the maximum power of the grid-connected PV systems for Morelia and Cuernavaca is 408.5kWp and 298 kWp, respectively.

Electric mobility could represent a decrease in the emission of polluting gases. For this, electrical energy must come from decarbonized sources. Since the Sun is a clean and inexhaustible energy source that can be use to generate electricity, we can propose a photovoltaic system to generate the required electricity. First we define the available surface and with this data calculate the possible PV energy production.

### Available surface for PV systems

With the help of satellite images we determine the available surface for PV systems in both cases. In the terminal of the GRAY ROUTE, the available surface limits the photovoltaic system sizing in Morelia, being around 5,646m^2^. In Cuernavaca city, there are two properties destined for bus garages. Station B available surface is 2,259.59m^2^, and another storage property is at the geographic coordinates (18.97440, -99.23644) with around 1,000m.^2^.

### Possible PV energy production

By using the equations described in the PV sizing section we calculate the possible energy production of the proposed photovoltaic systems.

**Fig 2:**
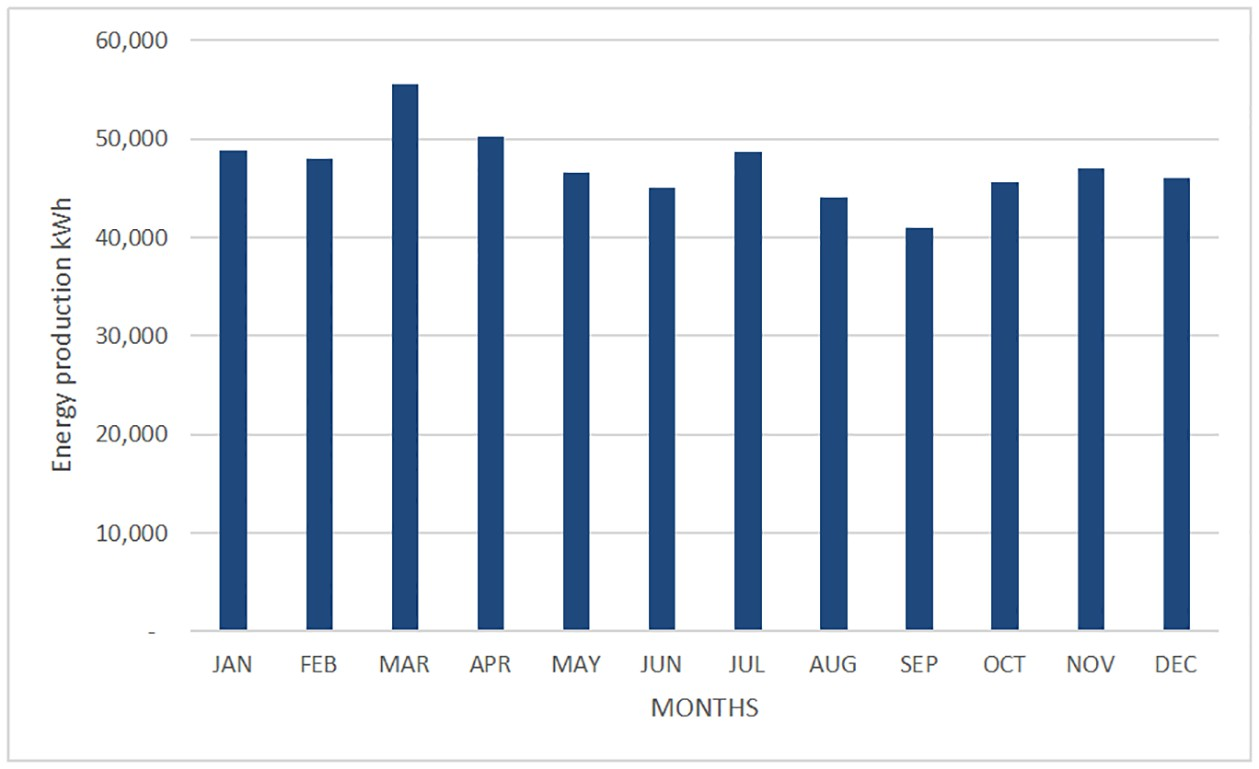
Monthly energy generated by the PV system in Morelia city.

**Fig 3:**
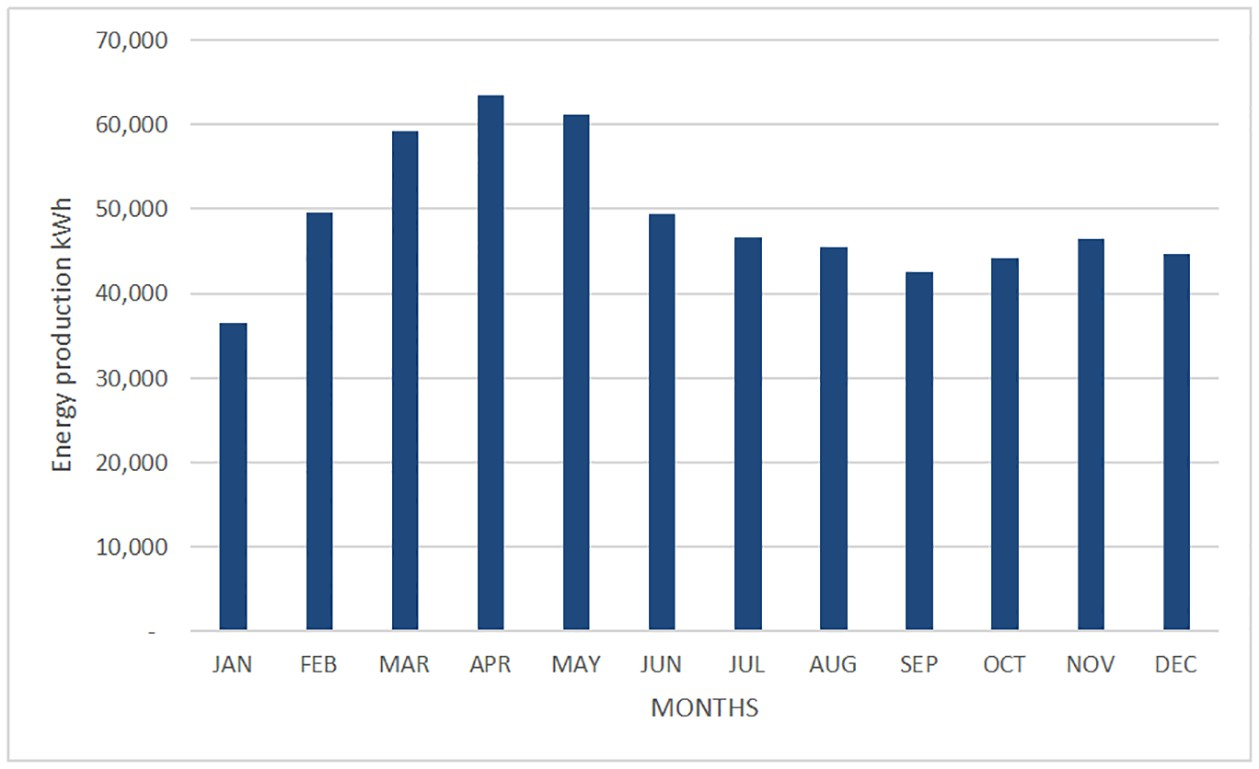
Monthly energy generated by the PV system in Cuernavaca city.

The energy production is shown in Table 8, Figs 2 and 3.

**Table 8:** PV System energy production.

Morelia city.				Cuernavaca city.		
*Month*	*R*_*S*_ (PSH)	*T*_*amb*_(°C)	PV(MWh)	*R*_*S*_ (PSH)	*T*_*amb*_ (°C)	PV(MWh)
*January*	3.4	26	36.52	6.28	28.3	48.84
*February*	5.16	28.2	49.56	6.93	31.3	48.02
*March*	5.66	31.8	59.22	7.31	33.5	55.52
*April*	6.3	32.6	63.55	6.85	33.9	50.25
*May*	5.88	32.9	61.21	6.15	33.9	46.62
*June*	4.83	29.4	49.44	6.03	33.9	45.05
*July*	4.41	29.4	46.64	6.27	28.7	48.67
*August*	4.29	28.8	45.50	5.69	29.3	44.05
*September*	4.41	28.6	42.53	5.46	28.7	41.02
*October*	4.16	28.3	44.22	5.84	27.6	45.56
*November*	4.49	26.9	46.48	6.23	27.8	46.99
*December*	4.16	26.2	44.64	4.16	26.2	46.32

The energy generated by the proposed PV system for each month of the year. The PV systems located in Morelia and Cuernavaca cities will produce per year around 589.5 MWh and 566.6 MWh respectively, this energy can reduce the energy demand coming from the grid.

In Morelia city, the period of greatest generation is from March to May; the lowest energy production takes place in September and January. Unlike the city of Morelia, the photovoltaic system located in Cuernavaca city has a more uniform power generation throughout the year. March is the highest power generation.

The annual distance traveled by the gray line in Morelia is 7,818,300 km, the energy demand to cover this distance is 2,293 MWh annually. Mercedes Benz buses demand 20,636 liters of Diesel annually, which represents an emission of 4,061 tons CO_2_, the annual energy required by 76 electric buses in Cuernavaca city is 1,065 MWh. The following table shows the CO_2_ annual emissions considering the fossil fuels, energy taken from the grid, and the PV supply.

The integration of a pv system contributes significantly to the emissions reduction.

**Fig 4:**
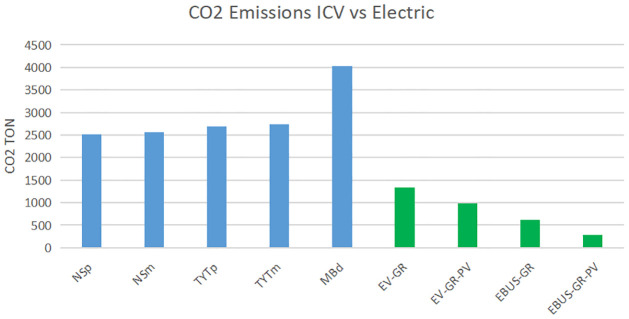
Comparison CO_2_ emissions ICV vs electric and PV alternatives for Morelia and Cuernavaca cities.

It is clearly observed (see Fig 4) that electric mobility reduces CO_2_ emissions, the implementation of photovoltaic energy also contributes significantly to the reduction of polluting emissions. In Fig 4 and Table 9, NSm, NSp, TYTm,TYTp, MBd denote Nissan Magna, Nissan Premium, Toyota Magnum, Toyota Premium and Mercedes Benz Diesel respectively. EV refers to the EV Van model E5 and Ebus refers to the electric bus Haojing.

**Table 9:** Annual CO2 emissions (TONs).

Morelia city.						Cuernavaca city.		
NSm	NSp	TYTm	TYTp	EV-Grid	EV-Grid-PV	Diesel	Ebus-Grid	Ebus-Grid-PV
2,565.65	2,521.87	2,730.59	2,683.99	1,335	991	4,023	619	290

Table 9 shows for the two cities case study the annual CO_2_ emissions from the ICV with the fuels currently used. It also show the annual CO_2_ emissions considering the replacement of the ICV by EVs with electricity supply only from the grid, and considering the energy contribution of the proposed photovoltaic systems.

### Energy costs

In this subsection, we present the energy costs for ICVs and EVs.

#### ICV fuel costs

We use fossil fuel prices and vehicles performance to calculate kilometer costs by applying Eq 1 and the economical considerations according to Table 10. In Table 11 the cost per kilometer for each vehicle and fuel are shown.

**Table 10:** Considerations in economic evaluation.

Morelia city.		Cuernavaca city.
Interest rate	10%	10%
Period (years)	25	25
ICV cost (USD)	$1,242,833	$1,702,838
Investment electric fleet (USD)	$1,890,000	$5,168,000
Energy demand (MWh)	2,293	1,065.21
Cost of energy (USD)	$310,050	$154,337
PV capacity (kW)	408.5	298.9
Investment PV systems (USD)	$604,580	$441,040
Energy produced (MWh)	589.5	566

**Table 11:** ICVs cost per kilometer.

Fuel prices and cost per kilometer.				
ICV	Fuel	Fuel Price (USD/l)	Performance (l/km)	Cost per km (USD/km)
Toyota	Magna	$1.03	0.149	$0.15
Toyota	Premium	$1.11	0.149	$0.16
Nissan	Magna	$1.03	0.140	$0.14
Nissan	Premium	$1.11	0.140	$0.15
Mercedes Benz	Diesel	$1.08	0.353	$0.38

Table contains the ICV cost per km according to the fuel type, current fuel prices and ICVs performance.

**Fig 5:**
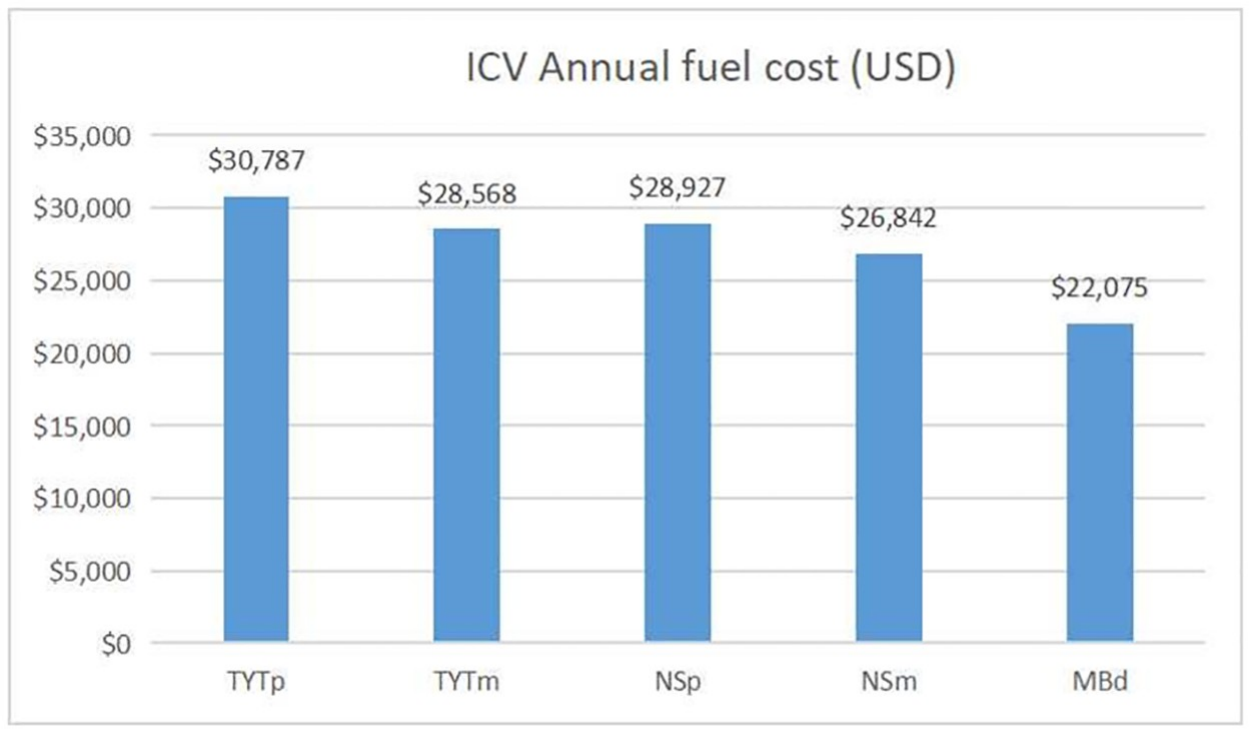
Annual fuel cost per vehicle, Morelia and Cuernavaca.

For Morelia city case average daily fuel cost is $76.5 USD, annually fuel cost average is $27,926.3 USD. Annual fuel costs are shown in Fig 5.

As can be seen in the graph, driving a Toyota Hiace vehicle using magna gasoline implies a cost of $28,568 USD per year and $28,927 for driving a Nissan Urvan using premium gasoline. Driving a Mercedes Benz bus in Cuernavaca city represents an annual cost of $22,075 USD.

#### EV electricity cost per km

According to the energy price in Mexico, using Eq 6 the EV cost per kilometer was calculated. Costs per km for both cities are shown in Table 12.

**Table 12:** EVs cost per kilometer.

Energy price and EV cost per kilometer.					
City	EV	Fuel	Energy Price	Performance	Cost (USD/km)
Morelia	EV van E5	Electricity	$0.07	0.29	$0.021
Cuernavaca	Ebus Haojing	Electricity	$0.07	0.24	$0.017

Table contains the EV cost per km in USD according to the HM tariff energy price (USD/kWh), EV performance is expressed in kWh/km.

#### Comparison ICV vs EV costs

Electric Vehicles (EVs) have fewer moving parts than internal combustion vehicles (ICVs); they do not need oil changes or filters. The powertrain of an EV is simpler than that of an ICV. Due to this simplicity, the EVs have 20-30% lower maintenance costs compared with ICVs [50, 51]. A cost comparison between ICV and EV is made, as we showed in Fig 5.

**Fig 6:**
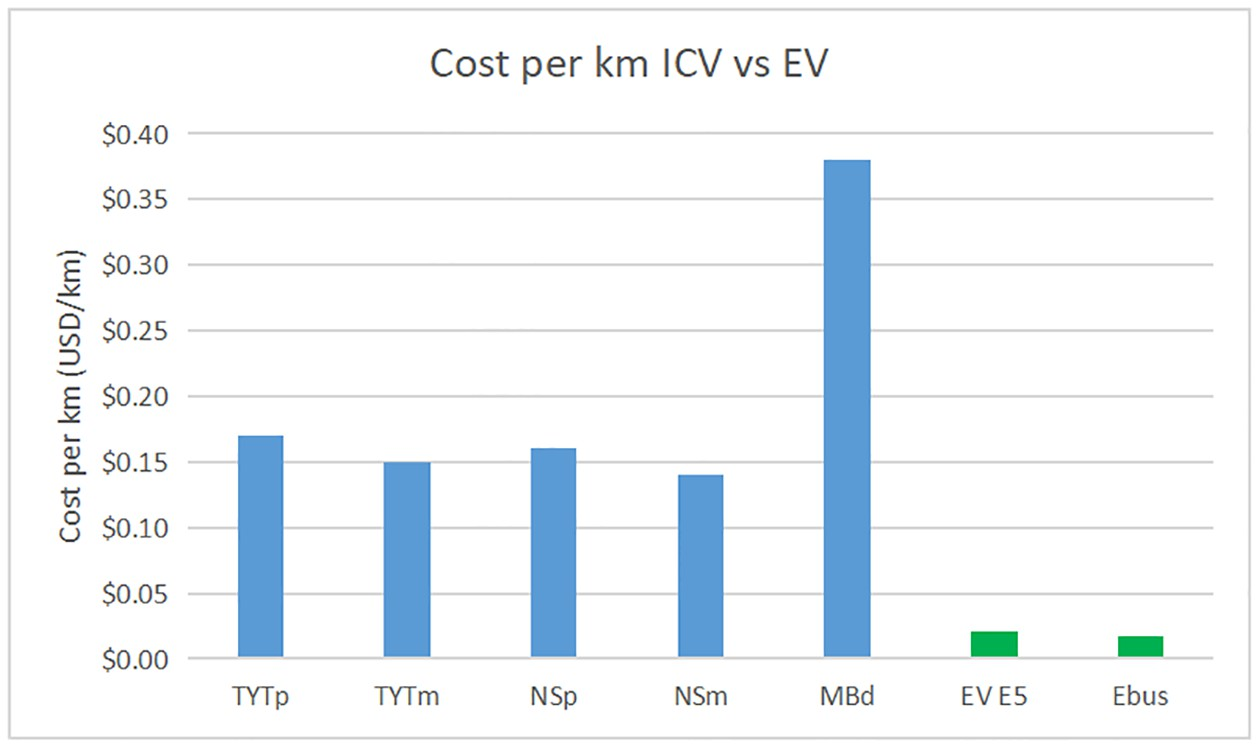
ICV vs EV cost per km.

**Fig 7:**
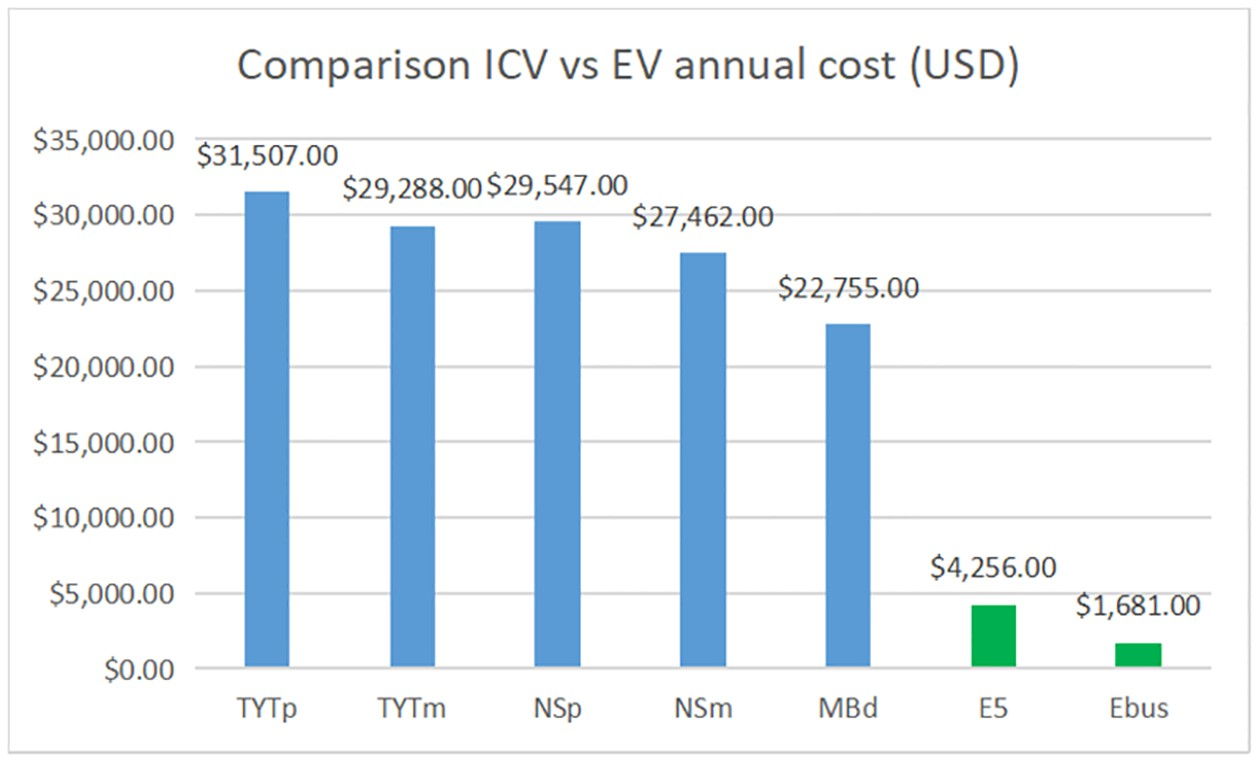
ICV vs EV annual cost.

A costs comparison between ICV and EV is done, the results are shown in Figs 6 and 7 and Table 13.

**Table 13:** Cost per km ICV vs electric alternative proposed.

Comparison ICV vs EV costs per km.		
Vehicle	Fuel	Cost per km (USD/km)
Toyota	Magna	$0.15
Toyota	Premium	$0.16
Nissan	Magna	$0.14
Nissan	Premium	$0.15
Mercedes Benz	Diesel	$0.38
EV van E5	Electricity	$0.021
Ebus Haojing	Electricity	$0.017

Current ICV cost per km vs electric mobility proposed.

Figs 6 and 7 show a comparison between the cost per kilometer with ICVs (Toyota Hiace, Nissan Urvan, Mercedes Benz Boxer) and proposed electric vehicles (E5 electric van and Ebus Haojing). This comparison shows the economic benefits of electric mobility. The cost per kilometer using the electric van Shiyan E5 and Ebus Haojing is cheaper than using the Nissan Urvan, Toyota Hiace, and Mercedes Benz bus. The cheapest cost per kilometer is obtained with the energy price of the HM rate ($0.07USD/kWh). According to the figures, GRAY ROUTE and RUTA UNO will save a considerable amount per EV Shiyan E5 and Ebus in fuel and maintenance costs.

### Grid-EV and GRID-EV-PV system scenarios

This subsection presents the two different alternatives of having the energy to charge the EVs in the two cities. First, we present the cost of obtaining only the energy from the GRID, and second, the cost with a reduction of the electricity demand because of the PV systems installed on the terminals.

#### Grid-EV scenario

We evaluate the economic feasibility for the electric fleet considering two scenarios. The first scenario involves using energy from the grid, and the second one makes the economic analysis regarding a PV energy system generator.

In the Grid-EV scenario, we consider that all-electric fleets’ required energy comes from the grid. The price of energy in Mexico has to be taken into account as well as the initial cost of electric fleet investment, cost of energy, the annual interest rate is 10% and 25 years in this analysis.

#### Grid-EV-PV system scenario

**Fig 8:**
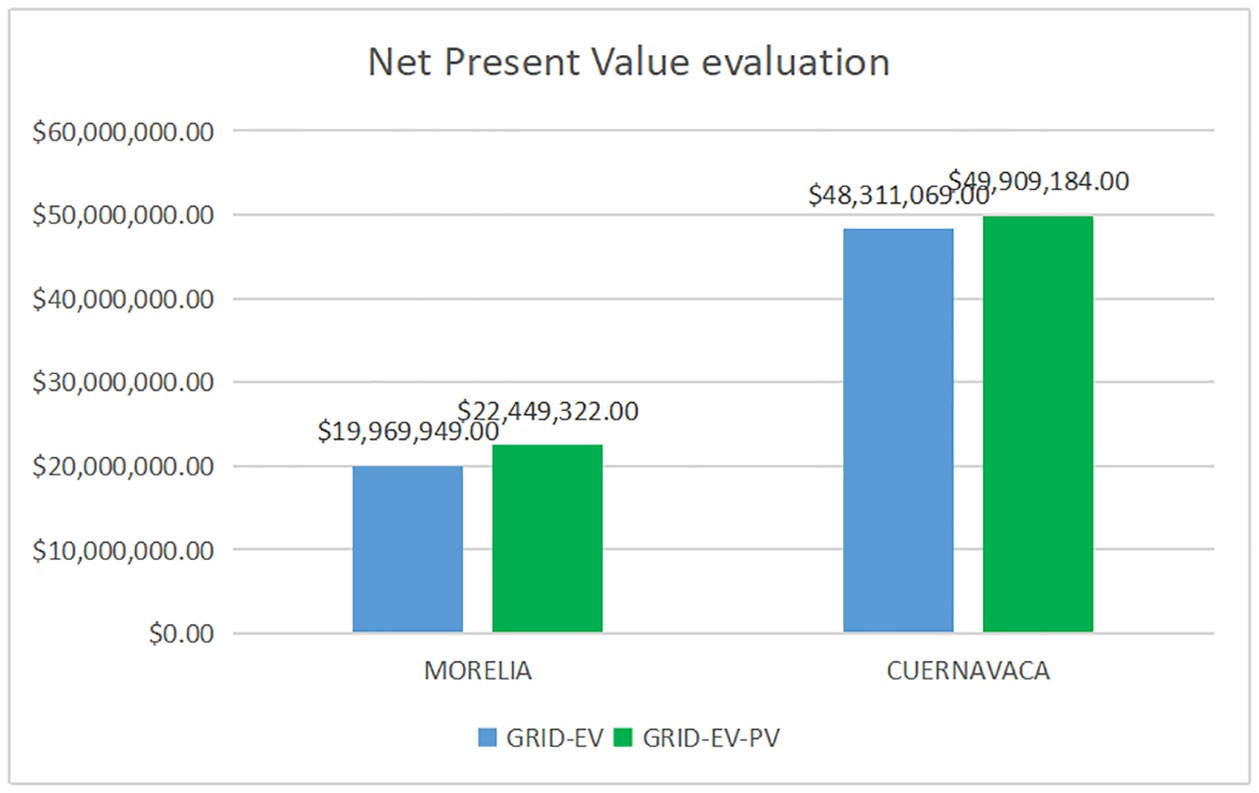
Net Present Value evaluation for two scenarios.

In this scenario, the energy produced by a PV system goes to the grid, thus, reducing the electricity demanded from the grid. PV system contributes 26.5% and 49% of the energy demand for Morelia and Cuernavaca city (see Fig 8), respectively. We regard the grid as a source of energy and unlimited storage. Results of the economic evaluation for both scenarios are in Table 14.

**Table 14:** Net Present Value evaluation for two scenarios.

Net Present Value.		
Scenario	Morelia NPV (USD)	Cuernavaca NPV (USD)
Grid-EV	19,969,949	48,311,069
Grid-EV-PV	22,449,322	49,909,184

The economic evaluation indicates that electric mobility in the two cities case study is viable for the two analyzed scenarios.

### Discussion

According to the latest IPCC report, scientists observed changes in the Earth’s climate in all regions and the climate system. Many of the observed climate changes are unprecedented in thousands, but in hundreds of thousands of years, some of the changes already occurring, such as continued sea-level rise, cannot be reversed for several centuries or millennia. However, a substantial and sustained reduction in carbon dioxide (CO_2_) and other greenhouse gas emissions would limit climate change. While improvements in air quality would be rapid, it could take 20 to 30 years for global temperatures to stabilize. That is why it is imperative to make a transition in transporting sector toward electric mobility. Of course, decarbonization in energy generation is vital. In Mexico, the energy matrix is based mainly on fossil fuels. It is necessary to move towards an energy policy that prioritizes energy generation from renewable sources. Renewable energy in conjunction with electrical mobility may decrease emissions associated with the transport sector.

According to the results obtained in this work, electric mobility is feasible in the two case study cities. From the economic point of view, it is cheaper to travel the same distances with an electric vehicle than with an internal combustion vehicle. From an environmental point, the transition to electric mobility would represent a decrease in carbon dioxide emissions. In addition, if photovoltaic systems are implemented in situ to supply energy to electric vehicles, the reduction of CO_2_ emissions would be even more significant. The results obtained in this work indicate that an electric public transport system in the two cities case study is viable from economic, technological, and environmental points of view. The economic analysis shows that even in the most expensive electric energy tariff, electric mobility is about 80% cheaper per kilometer than the current mobility by internal combustion vehicles. The most appropriate electric energy rate is HM because, at this rate, the cost of electric energy varies according to the time of day, which would make it possible to carry out the energy recharges of electric vehicles at the lowest cost of energy. For the case of the city of Morelia, the transition to electric public transport would reduce CO_2_ emissions about to 50%. For the city of Cuernavaca, these will decrease by 85%, considering the energy contribution of photovoltaic technology, emissions decrease 65% in the case of Morelia and 90% in Cuernavaca. Hence, the implementation of renewable energy is essential to achieve an even more significant emission reduction.

## Remarks

Many papers have addressed the issue of electric mobility, but most of them pay attention to Asian and European situations. There are not enough studies analyzing the benefits related to moving toward electric mobility for Mexico and other Latin American countries. This work shows the economic benefits of replacing internal combustion vehicles with electric vehicles and the additional environmental benefits of providing photovoltaic electricity. This study was carried out in two medium-sized cities in Mexico, Morelia, and Cuernavaca. We emphasize that the methodology proposed in this work can be applied to most cities in Mexico and Latin America. Currently, the public transportation system in Morelia and Cuernavaca uses fossil fuels for their operation. This internal combustion transport system implicitly entails environmental, economic, and social disadvantages. The transport sector is vulnerable to shortages and an increase in fuel costs, and environmental problems in both cities. It is necessary to design sustainable mobility alternatives that mitigate the adverse effects associated with the use of fossil fuels, such as CO_2_ emissions and pollutants. A viable alternative is the implementation of electric vehicles for public transport.

This work analyzes the possibility of moving towards a public transport system based on electric mobility. For this purpose, we used the methodology shown in Fig 1. First, we identify the type of ICV used in both cities, and we calculate the fuel costs and CO_2_ emissions associated with the operation of the public transportation system. Once we identify the mobility requirements, we select EV alternatives that could replace current ICVs. Subsequently, we determine the energy demand and the cost per km with EVs selected. We also propose the option of integrating a photovoltaic system that contributes to a fraction of the energy demand of electric vehicles and decreases CO_2_ emissions. Afterward, we make a comparison. Finally, we elaborate an economic analysis using the Net Present Value as a tool to evaluate the feasibility of an electric public transportation system.

We found that the transition to electric mobility brings convenient economical and environmental benefits. For the city of Morelia, electric mobility is 85% cheaper than currently mobility; moving one kilometer with currently ICVs costs $0.16 USD, while doing it with the proposed EV costs $0.02 USD. In the case of Cuernavaca city current Diesel bus cost per km is $ 0.38 USD; meanwhile, the Ebus cost per km is $0.017 USD. Thus, electric mobility is 95% cheaper than ICV mobility. As a result of the transition, we found a CO_2_ emissions reduction. Electric public transport could reduce annual CO_2_ emissions about 50%, from 2684 to 1335 CO_2_ (TONs) for Morelia and 85% from 4023 to 619 CO_2_ (TONs) for Cuernavaca city. In addition, the influence of photovoltaic technology contributes to a greater emissions reduction, for Morelia city emissions decrease annually 65% from 2684 to 991 CO_2_ (TONs) and 90% in Cuernavaca city from 4023 to 290 CO_2_ (TONs). Photovoltaic technology reduces the energy demand from the grid and decreases CO_2_ emissions. Therefore, it is imperative to move to electric public transport, taking into account renewable energy sources.

Mexico is located in the northern hemisphere and has a vast solar energy potential, 70% of its territory has average irradiation of 4.5 kWh/m^2^. Cuernavaca has an average solar resource of 6 kWh/m^2^ and the city of Morelia 4 kWh/m^2^. Thus, capturing and transforming this energy into electricity is possible to provide energy to electric vehicles. Photovoltaic technology has the characteristic of not requiring fossil fuels for electricity generation, there is no waste of any kind, so it is considered clean energy. Public transport is an ideal sector for the electrification of transport and integrating energy generation systems from renewable sources. The analysis shows the economic feasibility of replacing the ICVs that currently provide the public transport service in Morelia and Cuernavaca with efficient electric vehicles compatible with renewable energy sources.

We emphasize that the methodology proposed in this work can be applied to any other city.
